# Spatial analysis of neonatal near miss and socioeconomic and healthcare indicators in the state of Paraná

**DOI:** 10.1590/1980-549720250023

**Published:** 2025-05-09

**Authors:** Maria Luiza Melo da Silva, Natan Nascimento de Oliveira, Andreia Ferdin, Maria José Quina Galdino, Emiliana Cristina Melo, Rosana Rosseto de Oliveira

**Affiliations:** I Universidade Estadual de Maringá – Maringá (PR), Brazil.; II Universidade Estadual do Norte do Paraná – Bandeirantes (PR), Brazil.

**Keywords:** Near miss, healthcare, Indicators of morbidity and mortality, Infant, newborn, Information systems

## Abstract

**Objective:**

To analyze the spatial distribution of neonatal near miss and socioeconomic and healthcare indicators in the state of Paraná.

**Methods:**

Ecological, cross-sectional study of neonatal near miss rates in municipalities in the state of Paraná, from 2020 to 2022, obtained through data from the Live Birth Information System (SINASC) and the Mortality Information System (SIM), connected through deterministic linkage. The spatial distribution of neonatal near miss rates, socioeconomic indicators (maternal age and race/ethnicity), and healthcare indicators (type of delivery and number of prenatal consultations) were performed. Global and Local Moran's Index were used for spatial analysis.

**Results:**

The neonatal near miss rate in Paraná was 28.46 per thousand live births. Health regions (HR) 4^th^ HR - Irati, 3^rd^ HR - Ponta Grossa, 6^th^ HR - União da Vitória, and 17^th^ HR - Londrina stood out with high rates of neonatal near miss. Concerning the indicators, significant rates were evident among women of black, yellow, and indigenous race/color, as well as inadequacies in prenatal.

**Conclusions:**

The results highlight priorities in the Eastern and Northern macro-regions, where high-high clusters indicate an urgent need to assess access and quality of care. Additionally, there is a need to investigate neonatal near miss in Black, Yellow, and Indigenous women, as well as low prenatal adherence or coverage.

## INTRODUCTION

Across the world, significant progress has been achieved in reducing infant mortality^1^. However, challenges related to neonatal survival persist as a critical concern^2,3^.

Approximately 75% of infant deaths occur during the neonatal period (0 to 27 days), with the highest prevalence in the first week of life^2,3^. The majority of these deaths are attributable to preventable causes, including prematurity, low birth weight, congenital malformations, perinatal infections, and maternal factors^4,5^.

The quality of perinatal care is a crucial factor in mitigating maternal and neonatal morbidity and mortality^6^. Expanding knowledge about this process is equally essential, as complex cases offer valuable insights for more appropriate interventions, ultimately reducing the incidence of adverse outcomes^2^. In this context, the concept of neonatal near miss is increasingly recognized as a valuable tool for evaluating and enhancing the quality of care provided to the mother-child dyad, thereby contributing to the reduction of neonatal mortality^1^.

The term neonatal near miss refers to a newborn who, despite being at the brink of death, survives complications arising from pregnancy, childbirth, or the neonatal period^3^. Although this phenomenon is associated with the risk of predicting death, standardized or universally accepted criteria for its identification have yet to be established^7^.

The criteria for classifying a neonatal near miss case should be simple, practical, and easily applicable within the healthcare system, regardless of local conditions^8^. Moreover, these criteria must be closely associated with neonatal mortality, as both follow a similar trajectory, differing only in the final outcome^1,8^.

These cases offer extensive data for understanding the pathological processes that precede neonatal death, which are essential for evaluating healthcare performance, assessing the quality of care, and supporting interventions aimed at improving care for pregnant women, women in labor, and newborns^8,9^.

Furthermore, disparities in economic development, financial incentives, employment opportunities, education, and the expansion of the healthcare network contribute to barriers in service accessibility, with regional inequalities being particularly prominent^10^. Identifying the place of residence of neonatal near miss cases can generate essential local evidence for decision-making, as location is intrinsically associated with the availability and quality of healthcare services, as well as the demographic characteristics of the population^11^.

Given the significant disparities among different regions and population groups, this study is justified by its objective to analyze the spatial distribution of neonatal near miss cases in relation to socioeconomic and healthcare indicators in the state of Paraná from 2020 to 2022.

## METHODS

This ecological, cross-sectional study analyzed neonatal near miss rates in the state of Paraná from 2020 to 2022, following the guidelines of the Strengthening the Reporting of Observational Studies in Epidemiology (STROBE) tool.

Paraná, located in the southern region of Brazil, borders São Paulo, Santa Catarina, Mato Grosso do Sul, Argentina, Paraguay, and the Atlantic Ocean. Covering an area of 199,198.98 km^2^ and comprising 399 municipalities, it is the fifth-largest economy in the country. The state is divided into 22 health regions (HR) and four macro-regions: east, west, northwest, and north^12^.

To define neonatal near miss, the pragmatic criteria proposed by Pileggi-Castro et al.^13^ were adopted. According to these authors, the criteria include risk factors at birth, such as an Apgar score <7 at the 5^th^ minute of life, a birth weight <1,750 g, or a gestational age <33 weeks. Unlike the original authors, this study extended the neonatal period to 27 days, aiming for a more comprehensive assessment of complications that may arise after the initial days of life, as most studies typically focus only on the early period. Thus, cases of neonatal near miss were defined as those that survived up to 27 days and presented at least one of the aforementioned conditions.

The data were obtained through deterministic linkage between the databases of the Live Birth Information System (*Sistema de Informações sobre Nascidos Vivos* – SINASC) and the Mortality Information System (*Sistema de Informações sobre Mortalidade* – SIM) provided by the 15^th^ HR of the state of Paraná. The data, available at the individual level in spreadsheets, were collected in May 2023. Linkage is a methodological technique that connects different information sources into a single record, unifying them through a common identifier present in the databases^14^.

The linkage process was conducted in four stages, using different unifying variables sequentially: live birth certificate number, mother's name, father's name, and newborn's name. This approach aimed to maximize pairings, accounting for incomplete or incorrect records. Microsoft Office Excel's search and reference functions were employed for this purpose. Records that could not be paired at this stage underwent manual review to identify potential matches with different spellings but referring to the same individual. The procedure was carried out year by year, resulting in a concatenated database with a 98.94% success rate in pairing. The years 2020 to 2022 were selected to ensure a quantitatively adequate and updated sample for analysis.

The cases of neonatal near miss were analyzed based on the following sociodemographic variables: race/color (white, black, yellow, and indigenous), maternal age (<20 years, 20 to 34 years, and 35 years old and older). Additionally, the analysis considered healthcare-related variables, including the route of delivery (vaginal or cesarean) and the number of prenatal consultations (none, one to six, and seven or more consultations).

Neonatal near miss rates were calculated by multiplying the number of neonatal near miss cases and live births in the same location and period by 1,000. The crude near miss rates were then smoothed to account for the spatial variability of the area data across neighboring municipalities. The local Moran index (LISA) was calculated to assess the degree of spatial autocorrelation of neonatal near miss in municipalities and identify spatial clusters, which may indicate clusters:

- high-high: municipalities with high rates of neonatal near miss surrounded by municipalities that also have high rates;
- high-low: municipalities with high rates of neonatal near miss surrounded by municipalities with low rates;
- low-low: municipalities with low rates surrounded by municipalities with low rates;
- low-high: municipalities with low rates that border municipalities with high rates.

The cartographic base of the state of Paraná is available online in shapefile format on the website of the Brazilian Institute of Geography and Statistics (https://www.ibge.gov.br/geociencias/organizacao-do-territorio/malhas-territoriais.html). Chloropleth maps were created to illustrate the distribution of neonatal near miss rates across the municipalities of Paraná.

To facilitate data interpretation, the territorial limits of RS were overlaid on the maps. The spatial distribution was presented according to the Jenks classification, with the maps rendered in a purple scale, where lighter shades represent lower rates and darker shades indicate higher rates. All figures were generated using R software version 4.1.1, QGIS version 3.16, and GeoDA version 1.18.

The research adhered to the guidelines and regulatory standards outlined in Resolution No. 466/2012 of the National Health Council, which establishes ethical criteria for research involving human subjects. Consequently, the ethical requirements for approval by the Permanent Committee on Ethics in Research Involving Human Beings at Universidade Estadual de Maringá were fulfilled, and the study received approval under opinion No. 5.989.974/2023.

## RESULTS

**Figure 1:**
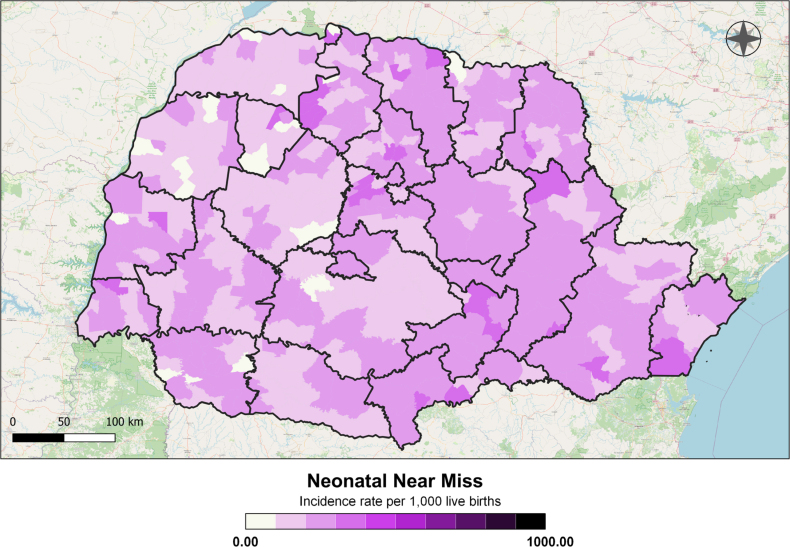
Distribution of neonatal near miss rates, by Health Regions and municipalities of residence, Paraná, Brazil, 2020 to 2022.

A total of 12,203 cases of neonatal near miss (28.46 cases per 1,000 live births — LB) were analyzed between 2020 and 2022. The spatial distribution revealed that the highest rates were unevenly distributed across the state, with notable concentrations in the municipalities of Florai, São Manoel do Paraná, Miraselva, and Paranapoema (69.62, 66.67, 61.22, and 60.98 cases per 1,000 LB, respectively). The lowest rates were observed in Jardim Olinda, Nova Aliança do Ivaí, Pinhal de São Bento, São Tomé, and Uniflor (Figure 1).

In the analysis by HR, a notable predominance of neonatal near miss cases was observed in Ponta Grossa, Irati, União da Vitória, and Londrina, with these HRs located in the eastern and northern macro-regions (Figure 1). Variations in rates were also observed among municipalities within the same RS, as seen in Maringá, where rates ranged from 0 to 69.62 per 1,000 LB (Figure 1).

**Figure 2:**
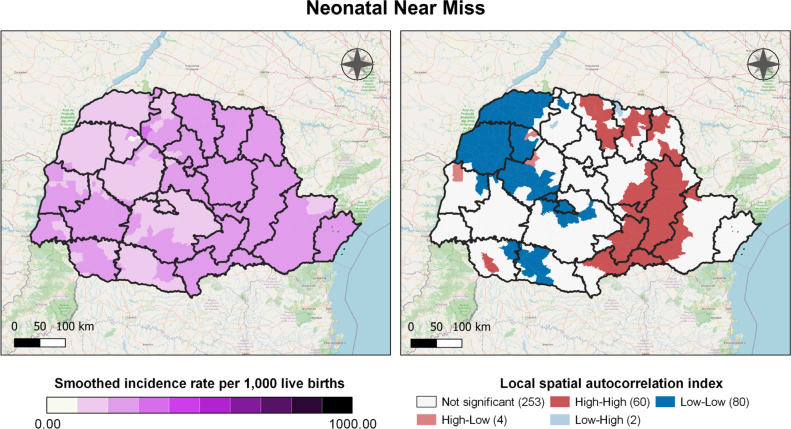
Smoothed rates of neonatal near miss and their clusters, Paraná, Brazil, 2020 to 2022.

By applying smoothing to the rates, this disparity was reduced, resulting in a more homogeneous distribution across the regions analyzed. The highest rates remained prominent in the HR located in the eastern and northern macro-regions (Paranaguá, Ponta Grossa, Irati, União da Vitória, Londrina, Cornélio Procópio, Jacarezinho, and Telêmaco Borba). Smoothing helped mitigate abrupt differences, emphasizing the general trends and revealing more consistent patterns in the distribution of the rates (Figure 2).

Regarding spatial autocorrelation, municipalities forming spatial clusters, known as high-high clusters, were identified (Figure 2). In this analysis, 60 municipalities exhibited a direct or positive spatial correlation, indicating high neonatal near miss rates in municipalities adjacent to others with similarly high rates. These municipalities are represented by two dark red clusters, the largest of which is located in the eastern and northern macro-regions, encompassing six health regions: Metropolitana, Ponta Grossa, Irati, União da Vitória, Jacarezinho, and Telêmaco Borba.

In contrast, 80 municipalities formed three clusters classified as low-low, indicating municipalities with low neonatal near miss rates surrounded by others with similarly low rates. These municipalities are represented in dark blue, with the largest cluster encompassing the regions of Guarapuava, Campo Mourão, Umuarama, Cianorte, Paranavaí, and Toledo (Figure 2).

Four municipalities were classified as high-low, indicating a negative or inverse spatial correlation. Similarly, two municipalities were classified as low-high (Figure 2).

**Figure 3:**
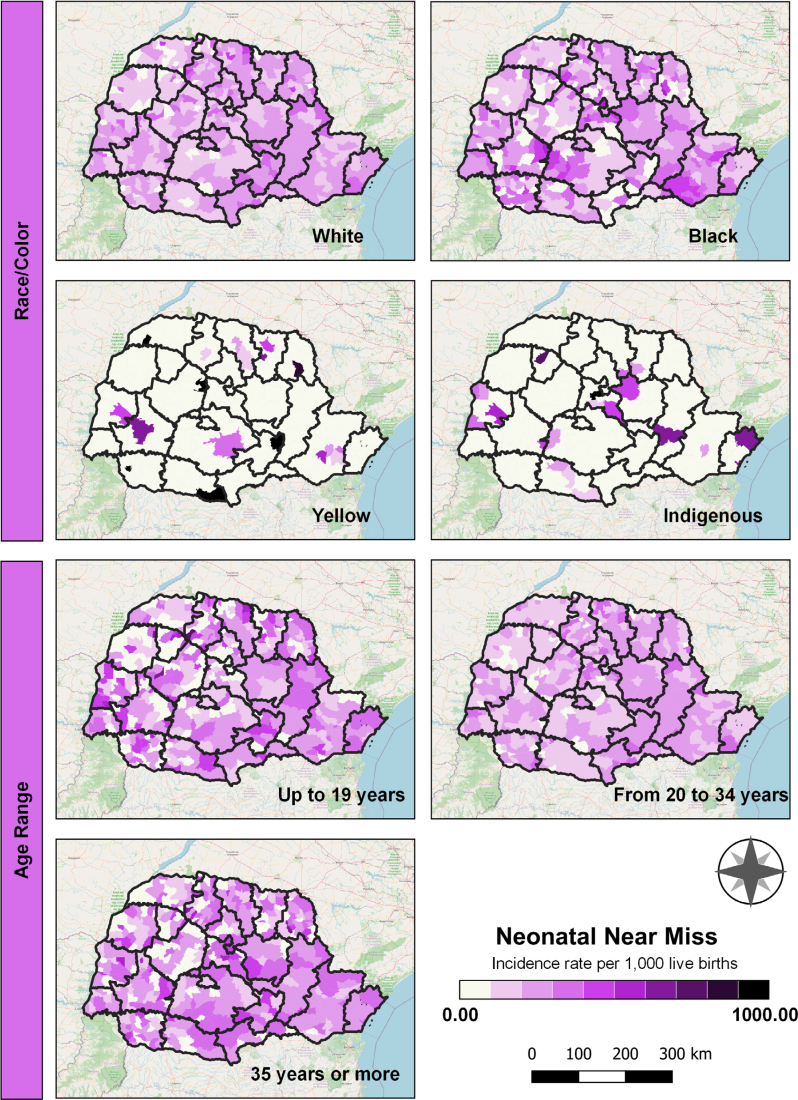
Spatial distribution of socioeconomic indicators, Paraná, Brazil, 2020 to 2022.

In socioeconomic indicators, high neonatal near miss rates were observed across most of the state among children of mothers who self-identified as Black. Elevated rates were also noted among children of mothers classified as Yellow in the municipalities of Teixeira Soares, Palmas, Santa Isabel do Ivaí, Barbosa Ferraz, Bela Vista da Caroba, and Tomazina, as well as among children of Indigenous mothers in Ivaiporã. Regarding maternal age, higher rates were observed among adolescent mothers (Figure 3).

**Figure 4:**
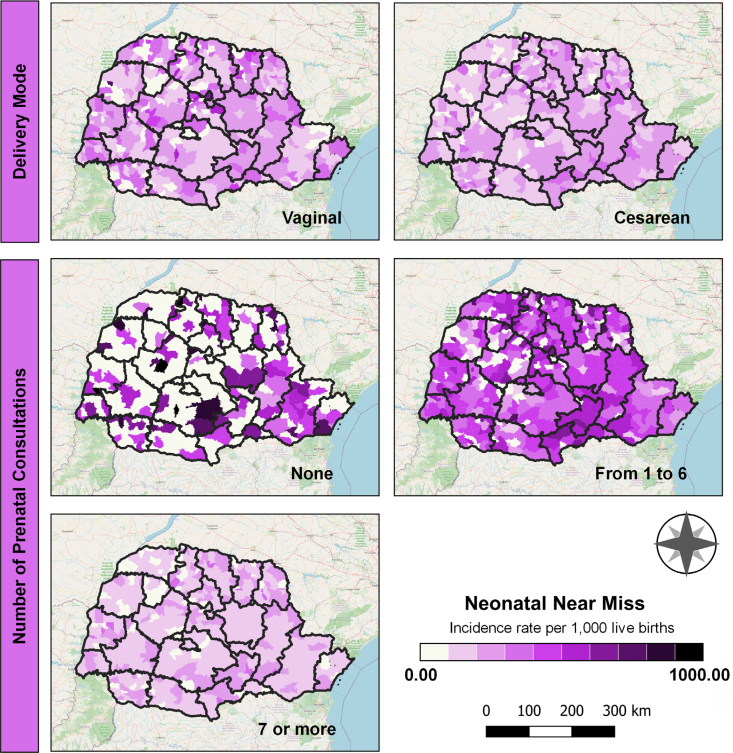
Spatial distribution of healthcare indicators, Paraná, Brazil, 2020 to 2022.

Regarding health care indicators, the highest neonatal near miss rates were observed among births by vaginal delivery, particularly in the municipalities of Esperança Nova, in the HR of Umuarama (northwest macroregion), and Cruzmaltina, in the HR of Ivaiporã (north macroregion). Conversely, cesarean sections were more prevalent in the eastern macroregion, particularly in the Metropolitan HR. Regarding the number of prenatal consultations and neonatal near miss occurrence, significant inadequacies were identified across much of Paraná. Many areas had an insufficient number of consultations, defined as fewer than seven, as recommended by the Paraná Maternal and Child Care Line (*Linha de Cuidado Materno Infantil do Paraná*)^15^. Cases in which no prenatal consultations were conducted were specifically observed in the eastern and northern macroregions, which also exhibited high rates of cesarean deliveries (Figure 4).

## DISCUSSION

This is the first study to adopt a spatial approach to neonatal near miss rates at the national level, providing insights into the relationship between the event and the geographic space in which it occurs. The findings allow for the identification of vulnerable regions, the interaction between neighboring municipalities, and the distribution of sociodemographic and healthcare indicators associated with the occurrence of neonatal near miss.

The neonatal near miss rate for the state was 28.46 per 1,000 LB, similar to rates observed in other regions of the country, such as Cuiabá (Mato Grosso – MT), with 22.8, and Joinville (Santa Catarina – SC), with 33 per 1,000 LB^7^. Despite these similarities, regional heterogeneity persists. A study conducted in Pernambuco reported a rate of 119.21 per 1,000 LB^16^, while another study in Ceará identified a rate of 54.11^7^. These findings reflect disparities across the Brazilian territory, associated with social, economic, and demographic factors, as well as challenges in timely access to health services^4,11^.

In this study, the municipalities of Florai, São Manoel do Paraná, Miraselva, and Paranapoema recorded the highest neonatal near miss rates. However, no cases of neonatal mortality were reported in these municipalities in 2022^18^. This may be associated with the quality of prenatal care, delivery, and neonatal care.

A greater discrepancy between the number of neonatal near miss cases and the number of deaths reflects the quality of care provided, indicating the ability of health services in these municipalities to prevent severe morbidity from resulting in fatalities^17^. Despite this apparent improvement in care, monitoring neonatal near miss remains essential, as its occurrence can lead to consequences of varying severity throughout an individual's life.

The damage caused by neonatal near miss can manifest immediately or later, including conditions such as sepsis, congenital infections, hemorrhages, impaired growth and development, cognitive and psychomotor changes, and an increased risk of chronic diseases such as cardiovascular diseases, stroke, diabetes, obesity, immune dysfunction, asthma, allergies, and others^19,20^. These effects can impact the quality of life of the child, the family, and health services.

Regarding the spatial autocorrelation of neonatal near miss, high-high clusters were observed in the eastern and northern macro-regions, raising questions about the efficiency of the Health Care Network (*Rede de Atenção à Saúde –* RAS), as these were the same regions with the poorest care indicators.

RAS is a fundamental instrument in organizing actions and services aimed at promoting and restoring health and preventing diseases, encompassing all levels of complexity. Its purpose is to ensure citizens’ access to health services based on their needs, guaranteeing the right to health and contributing to the reduction of inequalities^21^.

However, weaknesses in this model exist, such as communication failures between different points of the network. These issues are attributed to the lack of a shared information system across services of varying complexity levels and delays in performing and publishing test results, creating gaps in pregnancy monitoring^22^. These findings suggest the presence of system failures and highlight the need to restructure services in more densely populated areas, where demand for care is higher, as may be the case in the eastern macro-region.

The municipalities located in the northern macro-region, primarily colonized for agriculture and livestock farming, exhibit rural characteristics. With the exception of Londrina (with a population of over 400,000 inhabitants) and its metropolitan region, the other municipalities have small to medium-sized populations, rural and peripheral features, limited infrastructure, a shortage of specialized professionals, and high dependence on larger centers^23^. These characteristics hinder access to specialized health services, which are concentrated in medium and large municipalities.

Higher rates concentrated in macro-regions with distinct characteristics, such as the east and north, indicate the need for targeted public policies and greater attention to population extremes, both in regions with high population density and those with very low density. These disparities contribute to greater difficulty for pregnant women in accessing health services.

Regarding the distribution of socioeconomic and assistance indicators, the high rates of neonatal near miss among children of mothers who self-identify as Black, Yellow, or Indigenous corroborate findings in the literature that indicate a higher risk for these women^24^.

The vulnerability of Black pregnant women's health is widely recognized, highlighting difficulties in accessing the healthcare system, low quality of care, and a greater biological predisposition to certain conditions, such as high blood pressure and diabetes mellitus^25^. Women who identify as Asian, on the other hand, are at greater risk of premature births, while Indigenous newborns are more likely to be born with low birth weight compared to other races/colors^26^, characteristics indicative of neonatal near miss.

It is important to note that the state of Paraná has the 14^th^ largest Indigenous population in the country, with 54.41% living outside demarcated areas^27^. When migrating in search of better living conditions, this group encounters different, often hostile cultures, which can lead to significant invisibility in local public policies^28^. Although neonatal near miss rates for this population are already concerning, there is potential for even higher rates due to under-reporting of these categories, as Indigenous groups are often isolated within their cultural contexts^29^. This underscores the need for specific studies to further explore the context of this population.

Regarding maternal age, high rates of neonatal near miss are observed among adolescent mothers across HRs. In line with this, a study involving four birth cohorts, which aimed to investigate the association between sociodemographic factors, lifestyle, maternal reproductive profile, and prenatal and delivery care with neonatal near miss morbidity, identified an association between maternal age under 20 years and neonatal near miss^30^.

Both biological and social factors contribute to the risks associated with teenage pregnancy. Biological factors may include the physical and emotional immaturity of the adolescent body, while social factors may include limited access to adequate prenatal care, lack of family support, and insufficient sexual and reproductive health education^31^.

Most adolescents face additional challenges regarding prenatal care compared to adult women. They often start medical follow-up later and attend fewer consultations than recommended, sometimes due to the difficulty of coping with an unplanned pregnancy^32^. This increases the risk of adverse events.

Regarding the mode of delivery, a predominance of cesarean sections was observed in the eastern macro-region, particularly in the Metropolitan HR, where Curitiba, the state capital and one of the wealthiest cities in the country, is located^33^. It is well established that wealthier regions tend to have the highest prevalence of cesarean sections, which may be associated with profit motives and the perception of elective cesarean sections as a consumer product^9^. However, it is important to note that this is not a characteristic exclusive to the eastern macro-region, as the prevalence of cesarean sections is observed throughout the state (as illustrated in Figure 4). Paraná ranks fourth among Brazilian states with the highest incidence of cesarean sections, with an approximate rate of 63%^34^, much higher than the 15% recommended by the World Health Organization for the total number of births^35^.

Regarding the distribution of care indicators, a low number of prenatal consultations was observed in the occurrence of neonatal near miss throughout the state, corroborating studies that show an insufficient number of consultations during pregnancy is associated with neonatal near miss^7,24^.

It is important to note that prenatal care allows for the monitoring of pregnancy and fetal development, increasing the chances of detecting and intervening early in possible maternal and fetal complications^7,9^. However, meeting the recommended number of appointments alone does not guarantee quality care. It is essential to ensure that prenatal care begins early, ideally by the 12^th^ week of pregnancy, with qualified professionals, particularly nurses, whose training focuses on health promotion, disease prevention, and humanization. Additionally, the necessary resources must be available for adequate monitoring^4,24^.

Despite the limitations of this research, which was conducted with secondary data subject to incomplete information, underreporting of cases, and the inability to make direct causal inferences, it is emphasized that the study provides valuable and essential insights to support analyses and conclusions, enriching the understanding of the phenomenon under investigation.

The analysis of spatial distribution revealed priority areas, particularly the health regions of Irati, Ponta Grossa, União da Vitória, and Londrina, which exhibited high rates of neonatal near miss. Two high-high clusters were identified, with the largest located in the eastern and northern macro-regions, which also stood out for the prevalence of cesarean sections and inadequate prenatal care. This highlights the urgent need to assess both access to and the quality of care. Concerning sociodemographic indicators, significantly higher rates of neonatal near miss were observed among women who self-identified as black, yellow, or indigenous, as well as those with inadequate prenatal care. Further investigations are recommended to better understand these disparities. Such studies could inform strategies to improve maternal and child health indicators, including expanding access to prenatal care, enhancing monitoring through electronic systems, home visits, and awareness campaigns, as well as ensuring the provision of humanized care.

Recognizing regional differences is essential to develop targeted actions to prevent and reduce neonatal near miss, focusing on the specific needs of each area. These actions should include health education on family planning, contraceptive methods, and pregnancy, alongside ensuring a minimum number of prenatal consultations and individualized, high-quality care. This approach is a fundamental component in supporting pregnant women and preventing undesirable outcomes.

## References

[1] Tekelab T, Smith CCR, Loxton D (2020). Incidence and determinants of neonatal near miss in south Ethiopia: a prospective cohort study. BMC Pregnancy and Childbirth.

[2] Carvalho OMC, Xavier ATO, Gouveia APM, Augusto MCC, Carvalho FHC (2019). Identificação de casos de near miss neonatal: que critérios são usados no cenário brasileiro-revisão integrativa. Rev Med UFC.

[3] Brasileiro ALM, Rafael EV, Santos MH, Costa MS, Rabelo PKT, Vasconcelos YGR (2021). Morbidade neonatal near miss em um serviço de perinatalogia. Nursing.

[4] Silva AC, Migoto MT, Souza SJP, Tomin LL (2019). Indicadores de mortalidade perinatal, infantil e materna regional de saúde do estado do Paraná. Rev Gestão Saúde [Internet].

[5] Bernadino FBS (2022). Tendência da mortalidade neonatal no Brasil de 2007 a 2017. Ciênc Saúde Coletiva.

[6] Mario DN, Rigo L, Boclin KLS, Malvestio LMM, Anziliero D, Horta BL (2019). Qualidade do Pré-natal no Brasil: Pesquisa nacional de 2013. Ciênc Saúde Colet.

[7] Modes PSSA, Gaíva MAM, Andrade ACS, Guimarães LV (2023). Fatores associados ao near miss neonatal em uma capital do Centro-Oeste do Brasil. Rev Bras Saúde Mater Infantil.

[8] Maia MRG, Ferrari RAP, Cardelli APM, Higarashi IH, Carvalho MDB, Pelloso SM (2020). Near miss neonatal em unidade de terapia intensiva. Rev Bras. Enferm.

[9] Silva MLM, Moroskoski M, Back IR, Pereira MA, Oliveira RR (2024). Tendência das taxas de near miss neonatal e fatores associados no estado do Paraná. Rev Contrib Ciencias Sociales.

[10] Prezotto KH, Bortolato-Major C, Moreira RC, Oliveira RR, Melo EC, Silva FRT (2023). Mortalidade neonatal precoce e tardia: causas evitáveis e tendências nas regiões brasileiras. Acta Paul Enferm.

[11] Migoto MT, Oliveira RP, Andrade L, Freire MHS (2020). Correlação espacial da mortalidade perinatal com condições sociais, econômicas e demográficas: estudo ecológico. Rev Saúde Pública.

[12] Instituto Paranaense de Desenvolvimento Econômico e Social (IPARDES) (2021). Paraná em números [Internet].

[13] Pileggi-Castro C, Camelo JS, Perdoná GC, Mussi-Pinhata MM, Cecatti JG, Mori R (2014). Development of criteria for identifying neonatal near-miss cases: analysis of two WHO multicountry cross-sectional studies. BJOG.

[14] Queiroz OV de (2009). A construção da base nacional de dados em terapia renal substitutiva (TRS) centrada no indivíduo: relacionamento dos registros de óbitos pelo subsistema de autorização de procedimentos de alta complexidade (Apac/SAI/SUS) e pelo sistema de informações sobre mortalidade (SIM)-Brasil, 2000-2004. Epidemiol Serv Saúde.

[15] Paraná (2022). Secretaria de Estado da Saúde do Paraná. Linha de Cuidado Materno Infantil: Gestação [Internet].

[16] França KEX, Vilela MBR, Frias PG, Chaves MA, Sarinho SW (2021). Near miss neonatal em hospitais de referência para gestação e parto de alto risco: estudo transversal. Cad Saúde Pública.

[17] Carvalho OMC, Viana AB, Augusto MCC, Xavier ATO, Gouveia APM, Lopes FNB (2020). Fatores associados ao near miss e o óbito neonatais em maternidade pública de referência. Rev Bras Saúde Mater Infant.

[18] Brasil (2023). Departamento de Informática do SUS. Sistema de Informações sobre Mortalidade [Internet].

[19] Barbosa JMA, Brito AP, Reis AL, Rocha PG, Reis MAS, Araujo AP (2020). Prevalência de excesso de peso em crianças menores de dois anos e fatores associados. Revista Cereus.

[20] Pitilin EB, Ros GFD, Hanauer MC, Kappes S, Silva DTR, Oliveira PP (2021). Fatores perinatais associados à prematuridade em unidade de terapia intensive neonatal. Enferm.

[21] Schiller COA, Bellani WAGO, Moysés SJ, Werneck RI, Ignácio AS, Moysés ST (2021). Validação de face e construto do instrumento de avaliação de redes de atenção materno-infantil (IARAMI). Ciênc Saúde Coletiva.

[22] Soccol KLS, Marchiori MRT, Santos NO, Rocha BD (2022). Rede de atenção à saúde de gestantes e puérperas: percepções de trabalhadores da saúde. Saúde Coletiva.

[23] Dantas JS, Fajardo S, Silva FF (2023). A construção territorial do Paraná a partir da dinâmica econômica: considerações sobre as diferenças regionais.

[24] Pereira TG, Rocha DM, Fonseca VM, Moreira MEL, Gama SGN (2020). Fatores associados ao near miss neonatal no Brasil. Rev Saúde Pública.

[25] Born BH, Berroli CB, Luiz L, Baggenstoss MZ, Silva JC (2023). A influência da raça nos desfechos obstétricos. Braz J Health Rev.

[26] Matos LS, Assis TR, Sousa SR, Passos VM, Silva GF (2023). Recorte raça/cor dos indicadores materno-infantiis em uma regional de saúde do estado de Goiás. Rev Cient Estadual Saúde Pública de Goiás Cândido Santiago.

[27] Brasil (2023). Secretária da Comunicação. Censo 2022: Paraná tem 30.460 indígenas em 345 cidades [Internet].

[28] Tobias R, Cidade FC, Souza LC, Lima MCRF, Santos GRS, Mata LM (2023). Direito à cidade para povos indígenas na perspectiva do direito à saúde: uma revisão integrativa. Arq Urb.

[29] Carvalho D, Meirinho D (2020). O quesito cor/raça: desafios dos indicadores raciais de mortalidade materna como subsídio ao planejamento de políticas públicas em saúde. Rev Eletron Comum Inf Inov Saúde.

[30] Rocha PRH, Bettiol H, Confortin SC, Bazo G, Aristizábal LYG, Simões VMF (2022). Factors associated with neonatal-near miss: birth cohorts in three Brazilian cities - Ribeirão Preto, Pelotas and São Luís, Brazil. Ciênc Saúde Coletiva.

[31] Vieira AM, Santos DGS, Guimarães TMM (2020). Fatores que interferem na assistência ao pré-natal de gestantes adolescentes. Res Soc Dev.

[32] Assis TSC, Martinelli KG, Gama SGN, Neto ETS (2022). Fatores associados ao near miss neonatal em recém-nascidos de adolescentes brasileiras. Rev Esc Enferm USP.

[33] Brasil (2023). Secretária da Comunicação. Oito municípios paranaenses integram a lista das 100 maiores economias do país.

[34] Silva RL, Scudeler S, Leite BM, Santiago AC, Oishi RMO, Molina BM (2022). Análise sobre a taxa de cesáreas ocorridas na 15° regional de saúde do Paraná e um breve comparativo com a macrorregiões brasileiras. Braz J Dev.

[35] Prates LF de L, da Silva MLM, Moroskoski M, de Oliveira NM, Silva M de AP, Melo EC (2024). Tendência temporal e distribuição espacial das taxas das vias de parto, segundo características maternas no Brasil, entre 2011 e 2020. Rev Contexto Saúde.

