# Supplementary material for: Spatial analysis of neonatal near miss and socioeconomic and healthcare indicators in the state of Paraná
Source: Rev Bras Epidemiol. 2025 May 9;28:e250023. doi: 10.1590/1980-549720250023 (PMC12068812; doi:10.1590/1980-549720250023)
Supplement: Translation

# Translation: Análise espacial do *near miss* neonatal e indicadores socioeconômicos e assistenciais no estado do Paraná

**DOI:** 10.1590/1980-549720250023.2

**Authors:** Maria Luiza Melo da Silva, Natan Nascimento de Oliveira, Andreia Ferdin, Maria José Quina Galdino, Emiliana Cristina Melo, Rosana Rosseto de Oliveira

**Affiliations:** I Universidade Estadual de Maringá – Maringá (PR), Brasil.; II Universidade Estadual do Norte do Paraná – Bandeirantes (PR), Brasil.

**Keywords:** Near miss, Indicadores de morbimortalidade, Recém-nascido, Sistemas de informação

## Abstract

**Objetivo:**

Analisar a distribuição espacial do *near* miss neonatal e indicadores socioeconômicos e assistenciais no estado do Paraná.

**Métodos:**

Estudo ecológico e transversal das taxas de *near* miss neonatal nos municípios do estado do Paraná do período de 2020 a 2022, obtidas por meio de *linkage* determinístico do Sistema de Informações sobre Nascidos Vivos e do Sistema de Informações sobre Mortalidade. Foi realizada a distribuição espacial das taxas de *near miss* neonatal e dos indicadores socioeconômicos (idade materna e raça/cor) e assistenciais (via de parto e número de consultas pré-natais). Para a análise espacial foram empregados os índices de Moran global e local.

**Resultados:**

A taxa de *near miss* neonatal no Paraná foi de 28,46 por 1.000 nascidos vivos. As regionais de saúde Irati, Ponta Grossa, União da Vitória e Londrina se destacaram com altas taxas de *near miss* neonatal. Em relação aos indicadores, evidenciaram-se taxas expressivas em mulheres da raça/cor negra, amarela e indígena e a inadequação do pré-natal.

**Conclusões:**

Os resultados apontam prioridades nas macrorregiões leste e norte, onde há *clusters* alto-alto, evidenciando a urgência de se avaliarem o acesso e a qualidade da assistência. Além disso, destaca-se a necessidade de investigar o *near miss* neonatal em mulheres negras, amarelas e indígenas, bem como a baixa adesão ou cobertura do pré-natal.

## INTRODUÇÃO

Por todo o mundo, progressos notáveis foram observados a respeito da redução da mortalidade infantil^1^. Todavia, questões relacionadas à sobrevivência no período neonatal permanecem uma preocupação urgente^2,3^.

Cerca de 75% dos óbitos infantis ocorrem no período neonatal (0 a 27 dias), com maior prevalência na primeira semana de vida^2,3^. Esses óbitos ocorrem predominantemente por causas evitáveis, vinculadas a questões como prematuridade, baixo peso ao nascer, malformação congênita, infecções perinatais e fatores maternos^4,5^.

A qualidade da assistência perinatal é uma das ferramentas mais importantes para mitigar a morbimortalidade materna e neonatal^6^. Aprofundar o conhecimento sobre o processo é igualmente relevante, pois quadros complexos fornecem informações preciosas para condutas mais adequadas, reduzindo o número de desfechos indesejáveis^2^. Nesse contexto, o *near miss* neonatal está sendo cada vez mais aceito como ferramenta para avaliar e melhorar a qualidade dos serviços prestados ao binômio mãe-filho e, consequentemente, a redução da mortalidade neonatal^1^.

Entende-se que o termo *near miss* neonatal se refere ao recém-nascido que, em iminência de morte, sobrevive às complicações decorrentes da gestação, parto ou período neonatal^3^. Embora esse fenômeno esteja relacionado ao risco de predição da morte, ainda não há critérios padronizados nem universalmente aceitos para identificá-lo^7^.

Os critérios para classificar um caso de *near miss* neonatal devem ser simples, práticos e de fácil aplicabilidade no sistema de saúde, independentemente da realidade local^8^. Além disso, esses critérios devem estar estreitamente relacionados ao óbito neonatal, uma vez que seguem um trajeto semelhante ao da morte, diferenciando-se pelo desfecho^1,8^.

Esses casos fornecem uma vasta quantidade de dados para entender os processos de adoecimento que antecedem a morte neonatal, essenciais para monitorar o desempenho dos serviços de saúde, a qualidade do cuidado e apoiar ações de melhoria na atenção à gestante, à parturiente e ao recém-nascido^8,9^.

Ademais, é importante considerar que disparidades em desenvolvimento econômico, incentivos financeiros, oportunidades de emprego, educação e expansão da rede de assistência à saúde contribuem para fragilidades no acesso aos serviços, destacando-se as desigualdades regionais^10^. Identificar o lugar de residência dos casos de *near miss* neonatal pode fornecer evidências locais essenciais para decisões. Isso ocorre porque a localização está intrinsecamente ligada à disponibilidade e qualidade dos serviços de saúde e às características demográficas da população^11^.

Diante de um cenário marcado por significativas disparidades entre diferentes regiões e grupos populacionais, justifica-se este estudo, que teve como objetivo analisar a distribuição espacial do *near miss* neonatal e os indicadores socioeconômicos e de assistência no estado do Paraná, de 2020 a 2022.

## MÉTODOS

Estudo ecológico e transversal das taxas de *near miss* neonatal no estado do Paraná, no período de 2020 a 2022, conduzido pela ferramenta Strengthening the Reporting of Observational Studies in Epidemiology (STROBE).

O Paraná, localizado na Região Sul do Brasil, faz divisa com São Paulo, Santa Catarina, Mato Grosso do Sul, Argentina, Paraguai e o Oceano Atlântico. Com 399 municípios em 199.198,98 km^2^, é a quinta maior economia do país e divide-se em 22 regionais de saúde (RS) e quatro macrorregionais: leste, oeste, noroeste e norte^12^.

Para definir o *near miss* neonatal, foram adotados os critérios pragmáticos propostos por Pileggi-Castro et al.^13^. Para esses autores, esses critérios envolvem condições de risco ao nascer, como Apgar <7 no 5° minuto de vida, peso <1.750 g ou idade gestacional <33 semanas. Diferentemente dos autores originais, este estudo considerou o período neonatal até os 27 dias, visando a uma avaliação mais abrangente das complicações que podem surgir após os primeiros dias de vida, já que a maioria dos estudos foca apenas no período precoce. Portanto, foram considerados casos de *near miss* neonatal aqueles que não faleceram até 27 dias e apresentaram ao menos uma das condições referidas.

Os dados foram obtidos por meio do *linkage* determinístico entre as bases de dados do Sistema de Informações sobre Nascidos Vivos (SINASC) e do Sistema de Informações sobre Mortalidade (SIM) cedidos pela 15ª RS do estado do Paraná, em planilhas a nível individual, e coletados no mês de maio de 2023. *Linkage* é uma técnica metodológica que relaciona fontes de informações diferentes em um único registro, unificando-as por meio de um só identificador existente nos bancos de dados^14^.

O *linkage* foi conduzido em quatro etapas, utilizando diferentes variáveis unificadoras, sequencialmente: número da declaração de nascido vivo, nome da mãe, nome do pai e nome do recém-nascido. Esse processo visou maximizar os pareamentos, considerando registros incompletos ou incorretos. Para isso, utilizou-se uma das funções de pesquisa e referência disponíveis no *software* Microsoft Office Excel. Registros não pareados nessa fase foram submetidos a uma revisão manual para identificar possíveis correspondências com grafias diferentes, mas referentes ao mesmo indivíduo. O procedimento foi realizado ano a ano, obtendo-se um banco de dados concatenado, com 98,94% de sucesso no pareamento. Os anos de 2020 a 2022 foram selecionados com o intuito de obter-se uma amostra quantitativamente adequada e atualizada para a análise.

Os casos de *near miss* neonatal foram analisados levando-se em conta as variáveis sociodemográficas: raça/cor (branca, negra, amarela e indígena), idade materna (<20 anos, 20 a 34, e 35 e mais); e assistências: via de parto (vaginal ou cesáreo) e número de consultas de pré-natal (nenhuma, uma a seis, e sete ou mais consultas).

As taxas de *near miss* neonatal foram calculadas pela razão entre os casos de *near miss* neonatal e nascidos vivos do mesmo local e período, multiplicada por 1.000. Posteriormente, as taxas brutas de *near miss* foram suavizadas, considerando-se a variabilidade espacial dos dados de área, por meio de seus municípios vizinhos. O índice de Moran local (LISA, na sigla em inglês) foi calculado para avaliar o grau de autocorrelação espacial do *near miss* neonatal nos municípios e identificar aglomerados espaciais, podendo apontar *clusters*:

- alto-alto: municípios com altas taxas de *near miss* neonatal cercados por municípios que também possuem altas taxas;
- alto-baixo: municípios com índices elevados de *near miss* neonatal rodeados por municípios com índices baixos;
- baixo-baixo: municípios com baixas taxas rodeados por municípios com baixas taxas;
- baixo-alto: municípios com baixas taxas que fazem limite com municípios com altas taxas.

A base cartográfica do estado do Paraná está disponível *online* em *shapefile* no *site* do Instituto Brasileiro de Geografia e Estatística (https://www.ibge.gov.br/geociencias/organizacao-do-territorio/malhas-territoriais.html). Os mapas cloropléticos foram construídos para demonstrar a distribuição das taxas de *near miss* neonatal nos municípios paranaenses.

Para facilitar a interpretação dos dados, foi realizada a sobreposição com os limites territoriais das RS. A distribuição espacial foi apresentada conforme a classificação de Jenks, e elaboraram-se os mapas em escala de roxo, definindo cores mais claras para taxas mais baixas e cores mais escuras para taxas mais altas. Todas as figuras foram construídas usando os *softwares* R versão 4.1.1, QGIS versão 3.16 e GeoDA versão 1.18.

A pesquisa respeitou as diretrizes e normas regulamentadoras contidas na Resolução n° 466/2012 do Conselho Nacional de Saúde, que estabelece os critérios éticos para a realização de pesquisas envolvendo seres humanos. Assim, cumpriram-se os requisitos éticos de aprovação pelo Comitê Permanente de Ética em Pesquisa Envolvendo Seres Humanos da Universidade Estadual de Maringá, recebendo o parecer de n° 5.989.974/2023.

## RESULTADOS

Foram analisados 12.203 casos de *near miss* neonatal (28,46 casos de *near miss* por 1.000 nascidos vivos — NV), entre 2020 e 2022. A distribuição espacial demonstrou que as taxas mais elevadas se espalhavam de forma heterogênea pelo estado, destacando-se os municípios de Florai, São Manoel do Paraná, Miraselva e Paranapoema (69,62, 66,67, 61,22 e 60,98 casos por 1.000 NV, respectivamente). As menores taxas corresponderam a Jardim Olinda, Nova Aliança do Ivaí, Pinhal de São Bento, São Tome e Uniflor (Figura 1).

**Figura 1:** Distribuição das taxas de *near miss* neonatal, segundo Regionais de Saúde e municípios de residência, Paraná, Brasil, 2020 a 2022.

Na análise por RS, foi notória a predominância de casos de *near miss* neonatal em Ponta Grossa, Irati, União da Vitória e Londrina, com RS localizadas nas macrorregionais leste e norte (Figura 1). Observaram-se, ainda, variações nas taxas dos municípios de uma mesma RS, como na de Maringá, que apresentou municípios com taxas que variaram de 0 a 69,62 por 1.000 NV (Figura 1).

Ao aplicar a suavização nas taxas, viu-se redução nessa disparidade, tornando as taxas um pouco mais homogêneas ao longo das regiões analisadas, com ênfase para as RS localizadas nas macrorregionais leste e norte (Paranaguá, Ponta Grossa, Irati, União da Vitória, Londrina, Cornélio Procópio, Jacarezinho e Telêmaco Borba). A suavização ajudou a atenuar as diferenças abruptas, destacando as tendências gerais e revelando padrões mais consistentes na distribuição das taxas (Figura 2).

**Figura 2:** Taxas suavizadas do *near miss* neonatal e seus *clusters*, Paraná, Brasil, 2020 a 2022.

Em relação à autocorrelação espacial, foi possível identificar municípios que formaram conglomerados espaciais denominados de *cluster* (Figura 2). Nessa análise, 60 municípios apresentaram correlação espacial direta ou positiva classificada com *cluster* alto-alto, ou seja, municípios com alta taxa de *near miss* neonatal que eram vizinhos de outros que também apresentaram altas taxas. Esses municípios podem ser visualizados pela composição de dois *clusters* exibidos na cor vermelho-escura, sendo o maior localizado nas macrorregionais leste e norte, abrangendo seis regionais de saúde (Metropolitana, Ponta Grossa, Irati, União da Vitória, Jacarezinho e Telêmaco Borba).

Em contraste, 80 municípios compuseram três *clusters* com classificação baixo-baixo, ou seja, munícipios com baixa taxa de *near miss* neonatal circunvizinhos de outros que também apresentam baixas taxas de *near miss* neonatal. Esses munícipios foram apresentados na cor azul-escura, e o maior composto abrange Guarapuava, Campo Mourão, Umuarama, Cianorte, Paranavaí e Toledo (Figura 2).

Quatro munícipios foram classificados como alto-baixo, apresentando correlação espacial negativa ou inversa. Concomitantemente, dois munícipios foram classificados como baixo-alto (Figura 2).

Nos indicadores socioeconômicos, por grande parte do estado são observadas taxas elevadas de *near miss* neonatal entre crianças de mães que se declaram da raça/cor negra. Destacam-se também altas taxas entre crianças de mães da raça/cor amarela nos municípios de Teixeira Soares, Palmas, Santa Isabel do Ivaí, Barbosa Ferraz, Bela Vista da Caroba, Tomazina e indígenas em Ivaiporã. Quanto à idade, as taxas apresentaram-se mais altas para mães adolescentes (Figura 3).

**Figura 3:** Distribuição espacial de indicadores socioeconômicos, Paraná, Brasil, 2020 a 2022.

No que tange aos indicadores assistenciais, constatou-se que as taxas mais elevadas de *near miss* neonatal ocorreram entre os nascimentos por via de parto vaginal, sobretudo nos municípios de Esperança Nova, na RS de Umuarama (macrorregional noroeste), e Cruzmaltina, na RS de Ivaiporã (macrorregional norte). Em contrapartida, ficou evidente a ocorrência de cesarianas predominantemente na macrorregional leste, de maneira especial na RS Metropolitana. Com relação ao número de consultas pré-natais e à ocorrência de *near miss* neonatal, observou-se inadequação significativa em grande parte do território paranaense. Muitos locais tiveram um número insuficiente de consultas, ou seja, inferior a sete, conforme recomendado pela Linha de Cuidado Materno Infantil do Paraná^15^, incluindo casos em que nenhuma consulta foi realizada, especificamente nas macrorregiões leste e norte, destacando-se essas áreas também pelo elevado índice de partos cesáreo (Figura 4).

**Figura 4:** Distribuição espacial de indicadores assistenciais, Paraná, Brasil, 2020 a 2022.

## DISCUSSÃO

Este é o primeiro estudo a adotar uma abordagem espacial das taxas de *near miss* neonatal em todo o país, oferecendo contribuições significativas sobre a relação entre o evento e o espaço onde ocorre. As observações possibilitam identificar regiões vulneráveis, a interação entre municípios vizinhos e a distribuição dos indicadores sociodemográficos e assistenciais na ocorrência do evento.

A taxa estadual de *near miss* neonatal foi de 28,46 por 1.000 NV, semelhante à de outras regiões do país, como Cuiabá (MT), com taxa de 22,8, ou Joinville (SC), com taxa de 33 *near miss* por 1.000 NV^7^. Apesar das semelhanças, subsiste heterogeneidade entre regiões. Estudo realizado em Pernambuco identificou taxa de 119,21 *near miss* neonatal por 1.000 NV^16^, enquanto outro estudo realizado no Ceará identificou taxa de 54,1^17^. Tais observações evidenciam disparidades no território brasileiro, que podem ser atribuídas a questões sociais, econômicas, demográficas e ainda à dificuldade de acesso oportuno aos serviços de saúde^4,11^.

Neste estudo, os municípios de Florai, São Manoel do Paraná, Miraselva e Paranapoema registraram as maiores taxas de *near miss* neonatal, contudo não apresentaram casos de mortalidade neonatal no ano de 2022^18^, o que pode estar associado à qualidade do atendimento pré-natal, parto e período neonatal.

Quanto maior a discrepância entre o número de casos de *near miss* em relação ao número de óbitos, melhor é a qualidade do atendimento prestado, demonstrando a capacidade dos serviços de saúde desses municípios de evitar que casos de morbidade grave resultem em fatalidades^17^. Mesmo com essa aparente melhoria na assistência, é essencial conhecer a ocorrência de *near miss* neonatal, pois este pode gerar consequências de graus variados no decorrer da vida do indivíduo.

Os danos do *near miss* na vida do neonato podem ocorrer de forma imediata ou tardiamente, incluindo sepse, infecções congênitas, hemorragias, comprometimento no nível de crescimento e desenvolvimento, alterações cognitivas e psicomotoras, risco elevado para doenças crônicas como as cardiovasculares, acidente vascular cerebral, diabetes, obesidade, disfunção imunológica, asma, alergia e outras^19,20^, afetando a qualidade de vida da criança e da família e os serviços de saúde.

Quanto à autocorrelação espacial de *near miss* neonatal, observou-se a formação de aglomerados alto-alto nas macrorregionais leste e norte, o que leva a questionamentos acerca da eficiência da Rede de Atenção à Saúde (RAS), pois foram as mesmas regiões com piores indicadores assistenciais.

As RAS representam um instrumento fundamental na organização das ações e serviços voltados para promoção e recuperação da saúde e prevenção de doenças, abrangendo todos os níveis de complexidade. Seu propósito é garantir o acesso do cidadão aos serviços de saúde de acordo com suas necessidades, assegurando o direito à saúde e contribuindo para a redução das desigualdades^21^.

Todavia, há fragilidades nesse modelo, como falhas na comunicação entre os diferentes pontos da rede. Isso é atribuído à ausência de um sistema de informação compartilhado entre serviços de diferentes níveis de complexidade e à demora na realização e divulgação de resultados de exames, gerando lacunas no acompanhamento gestacional^22^. Essas constatações sugerem a existência de falhas e a necessidade de reestruturação dos serviços disponíveis em áreas mais densamente povoadas, onde a demanda por atendimento é mais expressiva, como pode ser o caso da macrorregional leste.

Já os municípios localizados na macrorregional norte, os quais tiveram sua colonização voltada expressivamente à agropecuária, possuem características de ruralidade. Com exceção de Londrina (com população superior a 400 mil habitantes) e sua região metropolitana, os demais municípios abrigam população de pequeno e médio porte, com características rurais e periféricas, carência de infraestrutura e profissionais especialistas e grande dependência de polos maiores^23^. Essas características dificultam o acesso aos serviços de saúde especializados, que se concentram em municípios de médio e grande porte.

As observações de maiores taxas concentradas em macrorregionais com distintas características, como a leste e norte, indicam a necessidade de políticas públicas e maior atenção aos extremos populacionais, em regiões tanto de alta densidade demográfica quanto de muito baixa densidade, as quais culminam com maior dificuldade de acesso das gestantes aos serviços de saúde.

No tocante à distribuição de indicadores socioeconômicos e assistenciais, as taxas elevadas de *near miss* neonatal para filhos de mães que se declaram de raça/cor negra, amarela e indígena corroboram achados da literatura que indicam maior risco para essas mulheres^24^.

A vulnerabilidade da saúde das gestantes negras é amplamente reconhecida, apontando para a dificuldade de acesso ao sistema, baixa qualidade do atendimento e maior tendência biológica para determinadas patologias, como hipertensão arterial e diabetes *mellitus* ^25^ *.* As mulheres que se declaram amarelas, por sua vez, apresentam risco para partos prematuros, enquanto os neonatos indígenas são os que mais nascem com baixo peso em relação às demais raças/cores^26^, características indicativas de *near miss*.

É importante destacar que o estado do Paraná possui a 14ª maior população indígena do país, com 54,41% vivendo fora de áreas demarcadas^27^. Ao migrar em busca de melhores condições de vida, esse grupo depara com culturas diferentes, muitas vezes hostis, o que pode convergir com significativa invisibilidade pelas políticas públicas locais^28^. Embora as taxas de *near miss* neonatal já sejam alarmantes para essa população, há possibilidade de taxas ainda mais altas, por causa do sub-registro dessas categorias, muitas vezes isoladas em seus contextos culturais^29^, determinando a necessidade de estudos específicos que se aprofundem no contexto dessa população.

Com relação à idade materna, observam-se taxas elevadas de *near miss* neonatal entre mães adolescentes distribuídas entre as RS. Em consonância, um estudo com quatro coortes de nascimento que teve como objetivo investigar a associação entre fatores sociodemográficos, estilo de vida, perfil reprodutivo materno e cuidados pré-natais e ao parto e a morbidade de *near miss* neonatal identificou associação entre idade materna inferior a 20 anos e o *near miss* neonatal^30^.

Existem razões tanto biológicas quanto sociais que contribuem para os riscos associados à gravidez na adolescência. Os fatores biológicos podem incluir a imaturidade física e emocional do corpo da adolescente, enquanto os fatores sociais podem incluir falta de acesso a cuidados pré-natais adequados, apoio familiar e educação sobre saúde sexual e reprodutiva^31^.

As adolescentes, em sua maioria, enfrentam desafios adicionais em relação ao cuidado pré-natal se comparadas às mulheres adultas. Elas com frequência começam o acompanhamento médico tardiamente e realizam um número menor de consultas do que o recomendado, por vezes em razão da dificuldade de lidar com uma gravidez não planejada^32^, aumentando o risco para eventos adversos.

No que tange à via de parto, observou-se a predominância do parto cesariano na macrorregional leste, principalmente na RS Metropolitana, na qual se localiza Curitiba, a capital do estado e uma das cidades mais ricas do país^33^. Sabe-se que as regiões mais ricas concentram as maiores prevalências de parto cesariano, o que pode estar associado ao lucro e à percepção da cesárea eletiva como um produto de consumo^9^. Ressalta-se, entretanto, que essa não é apenas uma particularidade da macrorregião leste, já que se observa a disseminação desse tipo de parto por todo o estado (conforme ilustrado na Figura 4), o qual ocupa a quarta posição na lista nacional de estados com maior incidência de cesarianas, registrando um índice aproximado de 63%^34^, valor muito superior ao 15% do total de partos preconizado pela Organização Mundial da Saúde^35^.

Ainda sobre a distribuição de indicadores assistenciais, observou-se baixo número de consultas de pré-natal na ocorrência de *near miss* neonatal por todo o estado, corroborando com estudos que evidenciam que o número insuficiente de consultas durante a gestação está associado ao *near miss* neonatal^7,24^.

Ressalta-se que o pré-natal possibilita acompanhar a gestação e o desenvolvimento fetal, favorecendo maiores chances de detecção e intervenção precoce de possíveis complicações maternas e fetais^7,9^. Todavia, o simples cumprimento do número recomendado de consultas não garante qualidade na assistência. É essencial assegurar o início precoce do pré-natal, idealmente até a 12ª semana de gestação, com profissionais qualificados, especialmente enfermeiros, cuja formação se pauta em promoção da saúde, prevenção de doenças e humanização, e contar com os recursos necessários para um acompanhamento adequado^4,24^.

Apesar das limitações desta pesquisa, realizada com dados secundários, sujeitos à incompletude de informações, à subnotificação dos casos e à impossibilidade de realizar inferências causais diretas, enfatiza-se que o estudo oferece *insights* valiosos e fundamentais para subsidiar análises e conclusões, enriquecendo o entendimento do fenômeno em estudo.

A análise da distribuição espacial permitiu identificar áreas prioritárias, destacando-se as RS Irati, Ponta Grossa, União da Vitória e Londrina, com altas taxas de *near miss* neonatal. Foram identificados dois *clusters* alto-alto, sendo o maior nas macrorregionais leste e norte, que também se destacaram pela ocorrência de partos cesáreos e pré-natal inadequado, evidenciando a urgência de avaliar o acesso e a qualidade da assistência. Quanto aos indicadores, observaram-se taxas expressivas de *near miss* neonatal entre mulheres autodeclaradas negras, amarelas e indígenas, bem como a inadequação do pré-natal. Sugere-se a realização de investigações específicas para compreender melhor esse cenário. Tais investigações podem subsidiar estratégias para melhorar os indicadores de saúde materno-infantil, incluindo a ampliação do acesso ao pré-natal e o monitoramento ativo das gestantes, via sistemas eletrônicos, visitas domiciliares e campanhas de conscientização, além de garantir assistência humanizada.

É necessário reconhecer as diferenças regionais para desenvolver ações de prevenção e redução desse evento, focadas nas necessidades locais, educação em saúde sobre planejamento familiar, métodos contraceptivos e gestação, além de garantir o mínimo de consultas de pré-natal e a qualidade individualizada do atendimento, um componente fundamental na assistência à gestante para prevenir eventos indesejáveis.
